# A novel device reduces anal pain after rubber band ligation: a randomized controlled trial

**DOI:** 10.1007/s10151-012-0824-7

**Published:** 2012-04-12

**Authors:** T. J. Lam, R. J. F. Felt-Bersma

**Affiliations:** Department of Gastroenterology and Hepatology, VU University Medical Center, P.O. Box 7057, 1007 MB Amsterdam, The Netherlands

**Keywords:** Rubber band ligation, Hemorrhoids, Anal pain, Hypothermia

## Abstract

**Background:**

Anal pain is a well-known sequel of rubber band ligation (RBL). A plastic device, the anal cooler which can be frozen in a freezer, has been developed to reduce anal pain. It contains a mixture of glycols and has a minimum temperature of 4 °C. This study was designed to investigate the efficacy of the anal cooler in pain relief after RBL.

**Methods:**

Between 2009 and 2010, 100 patients who underwent RBL were prospectively randomized into an anal cooler group (*n* = 50) or a control group (*n* = 50). The anal cooler group was instructed to use the cooler when they had pain. All patients were asked to keep a pain diary (0 = no pain; 10 = extreme pain), and follow-up was performed after 3–6 weeks.

**Results:**

It was found that 24/50 patients (48 %) in the anal cooler group and 31/50 (62 %) in the control group needed oral analgesics (NS). In total, 36/50 patients (72 %) used the anal cooler. Of these, 9/36 patients (25 %) noticed improvement. Of the remaining 27/36 patients (75 %) who did not notice improvement, 5/36 patients (14 %) found the insertion of the cooler uncomfortable and 1/36 patients (3 %) experienced nausea. No complications occurred during or after the use of the cooler. The 14/50 patients (28 %), who did not use the cooler, had a lower post-banding pain score compared with patients who used the cooler (1.4 vs 6.4; *P* < 0.001).

**Conclusions:**

Although post-banding pain after RBL is usually mild, the anal cooler seems to relieve anal pain in 25 % of the patients who used the device.

## Introduction

Symptomatic hemorrhoids are a common anorectal disorder [1, 2]. However, the exact incidence of this disease is unknown, since many individuals do not seek medical help. Studies evaluating the epidemiology of hemorrhoids have shown that the prevalence of hemorrhoids in the adult population is close to 4 % [3]. Several options are available for the treatment of symptomatic hemorrhoids and can be categorized into conservative medical management, non-surgical treatments and surgical techniques. Conservative medical management, including topical ointments and dietary modification with fiber or laxatives, is the first step in the treatment of patients with Grade I hemorrhoids [1]. Patients who have persistent symptoms or Grade II-III hemorrhoids may be candidates for minimally invasive non-surgical treatments, such as rubber band ligation (RBL), injection sclerotherapy, cryotherapy, infrared coagulation, laser therapy or diathermy coagulation [1, 4]. Surgical techniques are reserved for large symptomatic hemorrhoids that have not responded to conservative and non-surgical treatments [4]. Although surgical hemorrhoidectomy is more effective, it is usually associated with a higher complication rate [5, 6].

**Fig. 1:**
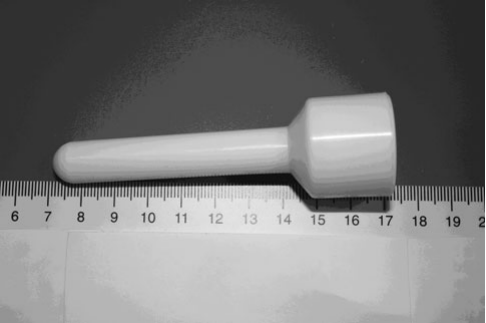
Anal cooler

Of all the non-surgical procedures, RBL seems to be the preferred first-line treatment for internal hemorrhoids [1]. This procedure has been recognized as safe, effective and easy to perform [7]. However, it is often associated with post-banding pain. In the literature, post-banding pain is documented with an incidence between 6 and 51 % [7–11]. In those situations, Sitz baths, mild analgesics and stool softeners are indicated. Unfortunately, the efficacy of those methods to alleviate pain is disappointing. A special device, the anal cooler, has been developed in an attempt to reduce anal pain (Fig. 1). The anal cooler, which can be cooled in the freezer, is a cylindrical-shaped plastic device containing a mixture of glycols and has a minimum temperature of 4 °C.

Application of cold has been used for many years as a non-pharmacological treatment for pain relief, particularly in acute soft tissue injury [12]. Benefits attributed to local cooling include reducing edema as a result of local vasoconstriction, slowing of cell metabolism, minimizing hemorrhage and decreasing the excitability of free nerve endings and peripheral nerve fibers, all of which result in an increased pain threshold [13, 14]. Therefore, it is reasonable to assume that the use of the anal cooler would be beneficial for anal pain. The aim of this study was to investigate the effectiveness and potential side-effects of the anal cooler in the relief of pain following RBL.

## Materials and methods

Between 2009 and 2010, 100 consecutive patients who were treated with RBL were included in the study. All patients had symptomatic hemorroids and normal colonoscopies. The patients were prospectively randomized into two groups: the anal cooler group (*n* = 50) and the control group (*n* = 50). Patients in the anal cooler group were instructed to keep the anal cooler in the freezer for at least 3 h prior to use and to use the anal cooler whenever they experienced anal pain for at least 10 min. If necessary they were allowed to use additional oral analgesics, such as paracetamol. The anal cooler was lubricated with vaseline and inserted into the anal canal and kept in place for up to 10–15 min (or for as long as it remained cold). The bulky part of the anal cooler remained outside the canal. Patients in the placebo/control group were also instructed to use oral analgesics when necessary. The patients kept a diary, which included a visual analog scale (VAS) regarding post-banding anal pain and use of oral analgesics where no pain was recorded as zero (0) and extreme pain as ten (10). All patients completed structured self-administered questionnaires regarding post-banding anal pain and analgesic requirements, and patient satisfaction was recorded. After 3–6 weeks, patients returned to the clinic for a new proctoscopic examination and evaluation of their diary. Ethics committee approval for the study was obtained from the Medical Ethical Commission of the VU University Medical Center (2009/19).

### Rubber band ligation

Rubber band ligation was performed in the standard manner. Patients were examined supine in the lithotomy position. The proctoscope was introduced into the anal canal allowing excellent visual control of the suction ligator. After suction started, the patient was asked whether he/she felt any pain. If the patient felt pain, suction was discontinued and the ligator introduced further until suction did not create any discomfort. Applications of the RBL were all 1–2 cm above the dentate line.

### The anal cooler

The anal cooler is a cylindrical-shaped plastic device 10 cm in length and 1 cm in diameter, provided by Lonnecker Medical, Enschede, the Netherlands. It contains a mixture of polyglycols and has a minimum temperature of 4 °C. Experimental studies in dogs have indicated that a similar device produces a fall of 10 °C in temperature of the rectal submucosa, with a return to the initial value in 7–10 min [15]. For use, the anal cooler was placed in the freezer. After 3 h, the anal cooler was then lubricated with vaseline and inserted into the anal canal and kept in place for up to 10–15 min (or for as long as it remained cold). The bulky part of the anal cooler remained outside the canal (Fig. 1).

### Statistical analysis

Results are presented as means and proportions. Differences between the mean pain levels were analyzed using Student’s *t*-test. Differences in the proportions were compared using Fisher’s exact test. Analyses were performed with the statistical software SPSS version 15.0 (SPSS Inc., Chicago, USA).

## Results

### Pre-treatment data

The mean age of the 100 patients was 54 years (range 22–88 years); there were 55 men and 45 women. The most frequent symptom at presentation was bleeding (*n* = 74). Other symptoms included prolapse (*n* = 64), anal pain (*n* = 33) and itching (*n* = 26). Thirty-three patients had undergone previous treatment**s** including RBL (*n* = 27), excisional hemorrhoidectomy (*n* = 4) and sclerotherapy (*n* = 1).

### Treatment data

**Fig. 2:**
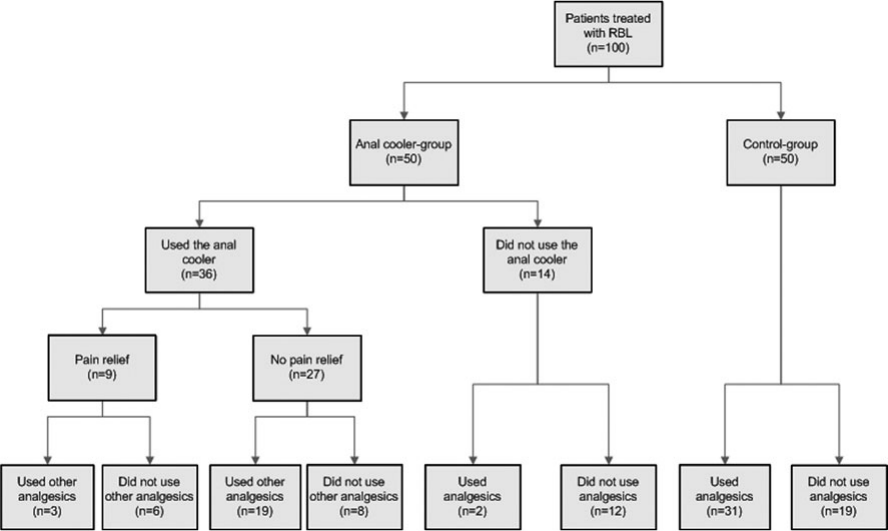
Flow-chart of 100 randomized patients

The mean number of bands applied per patient was 4 (range 1.8). After the treatment, 50 patients were randomized to the anal cooler group and 50 patients to the control group (Fig. 2). The demographics of both groups were not significantly different. The mean number of bands per session per patient was 1.6 (range 1–5).

### Post-treatment data

Fifty-four patients had a VAS-score ≥6. Nine of them sought medical advice prior to the planned follow-up visit. The mean VAS-score for post-banding anal pain on the day of the procedure was 5.5 and was similar in the anal cooler and the control groups (5.1 vs. 5.8; NS). Twenty-four of the fifty patients in the cooler group (48 %) and 31/50 patients (62 %) in the control group required oral analgesics to relieve pain (NS) (Table 1). Of these, 15/50 (30 %) and 18/50 (36 %) in the cooler and control groups, respectively, needed more than 2 analgesic tablets (NS). In total, 36/50 patients (72 %) used the anal cooler with 9/36 patients (25 %) reporting significant pain reduction. In these patients, the use of analgesics tended to be lower compared with patients who did not experience a symptomatic improvement with the cooler (33 % vs. 70 %, respectively: *P* = 0.11). In total, 3 patients, who noticed an improvement with the cooler, also used analgesics and 2 of these patients used pre-emptive analgesia due to fear of pain and 1 patient required additional analgesics on days 1 and 2 after RBL because the cooler was deemed ineffective. Of the remaining 27 patients (75 %), who did not have symptomatic improvement with the use of the cooler, 5 (14 %) found insertion uncomfortable and 1 (3 %) complained of nausea which was related to the insertion of the anal cooler.

**Table 1:** Characteristics of anal cooler group and control group

	Anal cooler group *n* = 50			Control group *n* = 50
	Pain relief with anal cooler *n* = 9 (18 %)	No pain relief with anal cooler *n* = 27 (54 %)	Not used *n* = 14 (28 %)	
Age				
Year (range)	55 (29–64)	49 (25–72)*^,¿^	57 (22–85)*	55 (30–88)^¿^
Gender				
Female (*n*)	3 (33 %)	10 (37 %)	8 (57 %)	24 (48 %)
Male (*n*)	6 (67 %)	17 (63 %)	6 (43 %)	26 (52 %)
History				
First treatment (*n*)	6 (67 %)	21 (78 %)	9 (64 %)	32 (64 %)
Recurrence after RBL (*n*)	3 (33 %)	5 (19 %)	4 (29 %)	15 (30 %)
Recurrence after sclerosis (*n*)	0	1 (4 %)	0	3 (6 %)
Recurrence after surgery (*n*)	0	0	1 (7 %)	0
Clinical presentation				
Bleeding (*n*)	8 (89 %)	21 (78 %)	11 (79 %)	34 (68 %)
Pruritis (*n*)	2 (22 %)	7 (26 %)	4 (29 %)	13 (26 %)
Pain (*n*)	3 (33 %)	14 (52 %)	2 (14 %)	14 (28 %)
Prolapse (*n*)	3 (33 %)	19 (70 %)	10 (71 %)	32 (64 %)
Defecation				
Frequency (mean)	1.6 per day	1.2 per day	1.6 per day	1.4 per day
Consistency				
Soft (*n*)	3 (33 %)	6 (22 %)	3 (21 %)	16 (32 %)
Normal (*n*)	6 (67 %)	18 (67 %)	9 (64 %)	21 (42 %)
Hard (*n*)	0	1 (4 %)	2 (14 %)	7 (14 %)
Variable (*n*)	0	2 (7 %)	0	6 (12 %)
Number of ligations				
Mean (range)	3.1 (1.3)^$^	4.0 (1.1)^$^	3.5 (1.4)	4.2 (2–8)
Total times of using cooler				
Mean	4.3	3.5	0	–
Post-banding pain (mean VAS-score)				
Day 0	5.1 (SD: 2.4)^&^	7.1 (SD: 2.6)^&,§,±^	1.4 (SD: 1.9)^§,@^	
Day +1	4.7 (SD: 2.1)^#^	6.6 (SD: 2.4)^#,¶,+^	0.6 (SD: 1.2)^¶,@^	
Day +2	1.7 (SD: 2.3)^^^	5.0 (SD: 3.2)^^,μ^	0.8 (SD: 1.5)^μ,@^	
Day +3	1.7 (SD: 2.2)^%^	4.6 (SD: 3.1)^%,†^	0.6 (SD: 1.1)^†,@^	
Pharmacologic therapy for pain (*n*)	3 (33 %)^¥^	19 (70 %)^≦,€,≤^	2 (14 %)^€^	31 (62 %)^≤^
RBL sessions per patient				
Mean (range)	1.4 (1–3)	1.4 (1–3)	1.9 (1–5)	1.5 (1–4)

* *P* = 0.07; ^@^ *P* < 0.001; ^%^ *P* = 0.02

^¿^ *P* = 0.03; ^#^ *P* = 0.04; ^†^ *P* < 0.001

^$^ *P* = 0.06; ^¶^ *P* < 0.001; ^¥^ *P* = 0.11

^&^ *P* = 0.052; ^+^ *P* = 0.005; ^€^ *P* = 0.06

^§^ *P* < 0.001; ^^^ *P* = 0.01; ^≤^ *P* = 0.002

^±^ *P* = 0.05; ^μ^ *P* < 0.001

The main complaint of patients who did not experience improvement with the anal cooler was that the cooler did not appear to be cold enough. Five minutes after insertion, the temperature of the anal cooler increased to body temperature. No complications occurred during or after the use of the anal cooler. The 14/50 patients (28 %) who did not use the anal cooler had a significantly lower post-banding VAS-score when compared with patients who used the anal cooler. Two of these patients needed pain medication and did not try the anal cooler, because of fear of pain during insertion.

### RBL complications

Besides post-banding pain, other complications included rectal bleeding (3 %), localized infection without abscess formation or fever (1 %), post-infection ‘polyp’ (1 %) and urinary retention (1 %). Two patients required hospitalization for rectal bleeding which was treated conservatively without the need for blood transfusion.

## Discussion

Anal pain is a well-known sequel of RBL. In this study, 54 % of the patients had a VAS-score ≥6 with 55 % requiring oral analgesics. The anal cooler relieved anal pain in 25 % of the patients. Its use tended to decrease the analgesic requirement; however, this effect failed to reach statistical significance due to the small sample size. The use of the anal cooler had no serious side-effects.

In the last few decades, local cooling has been used with some frequency in the management of acute local tissue injury, including perianal trauma as well as after minor surgical interventions and in the treatment anal fissure [16–19]. Furthermore, in small studies, local cooling has been suggested to relieve some of the symptoms of hemorrhoids [15, 20]. A reduction of the soft tissue temperature by 10 °C decreases local cellular metabolism, reducing edema by constriction of the peripheral blood vessels, as well as minimizing hemorrhage and diminishing the excitability of free nerve endings and peripheral nerve fibers; each of which results in an increase in the pain threshold [13, 14].

Notwithstanding these effects, the anal cooler was only effective in 25 % of the patients who used it consistently. In some cases, the potential benefit was mitigated by a reportedly painful insertion of the device. For a beneficial effect, an application time of at least 10–15 min is necessary and it is necessary that the patient continue to insert the cooler despite initial discomfort. In this respect, a beneficial effect may potentially occur if the anal cooler is lubricated with lidocaine gel rather than vaseline. The second most likely reason for the limited efficacy of the anal cooler was that it was not cool enough and that the time that the cooler stayed cold was probably too short. In this regard, it might be useful to add water to the mixture of the anal cooler so as to improve its freezing characteristics and reduce its temperature. The third reason for a relative lack of efficacy is the ease of use of the device. The anal cooler needs to be kept in the freezer and requires an application time of 10–15 min which may be inconvenient for working patients.

## Conclusions

In conclusion, the results of this prospectively randomized trial show that post-banding pain is usually mild and although there is no statistical advantage that there may be clinical benefit in the use of an anal cooler following RBL.
